# Accuracy and reliability of maxillary digital model (MDM) superimposition in evaluating teeth movement in adults compared with CBCT maxillary superimposition

**DOI:** 10.1038/s41598-020-76537-w

**Published:** 2020-11-09

**Authors:** Yichen Pan, Xin Wang, Fanfan Dai, Gui Chen, Tianmin Xu

**Affiliations:** 1 Department of Orthodontics, School and Hospital of Stomatology, 22# Zhongguancun Avenue South, Haidian District, Beijing, People’s Republic of China; 2 Second Dental Center, School and Hospital of Stomatology, 66# Anli Road, ChaoYang District, Beijing, People’s Republic of China

**Keywords:** Image processing, Software, Dental diseases

## Abstract

Superimposition of 3D maxillary digital dental models of different time points based on palatal vault region has been used to evaluate tooth movement during orthodontic treatment. This study evaluated the accuracy and reliability of 3D maxillary digital model (MDM) superimposition in adults by comparing it with CBCT maxillary superimposition. In CBCT maxillary superimposition, voxel-based superimposition was firstly conducted, and MDMs were matched with corresponding CBCT models (CBCT-MDM superimposition). MDM superimposition (palatal vault regional superimposition) were performed on another pair of pre- and posttreatment MDMs. The position and orientation of upper first molars (U6s) and upper central incisors (U1s) on the posttreatment MDMs were compared to assess the accuracy of the MDM superimposition methods. The reliability was validated in both MDM superimposition and CBCT maxillary superimposition. In terms of accuracy, the average linear deviations in U6 and U1 positions were less than ± 0.3 mm, the average angular deviations of U6s and U1s were less than ± 0.2°, both have no significant difference from zero. The ICCs for MDM superimposition ranged from 0.85 to 0.99. The ICCs for CBCT-MDM superimposition were larger than 0.99 in all items. MDM superimposition was an efficient, accurate and reliable method for evaluating teeth movement in adults, although its reliability is slightly lower than CBCT maxillary superimposition.

## Introduction

Superimposition of the pre- and posttreatment medical images is an important approach to evaluate the changes in hard and soft tissues, especially in tooth movements, during orthodontic treatment^1–4^. At first, superimposition of cephalometric radiographs was used to evaluate tooth movement and also had become the main source of knowledge of craniofacial growth and development. However, several limitations held up its further use. Two-dimensional cephalometric measurements can only reflect changes in sagittal and vertical direction, with the addition of the problems with image magnification and distortion^5,6^.

Another clinical material, dental model, was the first to be used in evaluating three-dimensional tooth movements because of its ability to accurately display the three-dimensional morphology of dental crowns. Many studies have been done to measure tooth movements on dental models^7–9^. The palatal rugae have been used as reference for direct measurement of anteroposterior tooth movement in the maxilla^10^. Not until the appearance of digital dental model had the real superimposition of dental models become possible. In region superimposition, part or whole palatal vault was usually used as the reference and the best-fit methods of various software were adapted^11,12^. Cha et al.^13^ were the first to evaluated tooth movements during treatment through digital dental superimposition who used the whole palatal vault as the reference for superimposition. Choi et al.^11^ used the whole palatal region from the first palatal rugae to the distal surfaces of bilateral maxillary second molars, and the lateral margin extended to 5 mm away from the gingival margin of posterior teeth. Jang et al.^14^ used anchorage miniscrews to explore the palatal stable region. Different from his counterpart, Chen et al.^12^ used unloaded miniscrews and established a palatal vault region for superimposition including the medial 2/3 of the third rugae and the regional palatal vault dorsal to it whose change were less than 0.5 mm after treatment, smaller than the reference region described by Choi et al.^11^. Vasilakos et al.^7^ have found that this area superior than other smaller reference regions and the region raised by Choi et al.^11^. However, stable region of the palatal structure is soft tissue which entails constant validation of its use in superimposition.

The wide use of cone beam computed tomography (CBCT) have made it possible for clinicians to superimpose the images taken at different time points on three-dimensional structures. In CBCT images, we can obtain three-dimensional data at a 1:1 ratio with the real anatomic structures. As a tool to evaluate tooth movement, the advantages of CBCT lie in that bone structures can be used as reference for superimposition and, at the same time, the dental roots are visible in three dimensions. Recently raised voxel-based superimposition method align two CBCT images using mathematical algorithms based on the greyscale values, not affected by 3D reconstruction compared with surface superimposition^15–17^. However, dental crown area, especially the occlusal surface, was not as accurate and clear in reconstructed CBCTs as in digital dental models. Furthermore, taking CBCT scans will expose the patients to extra radiation dose. On the other hand, radiation-free dental models can be obtained repeatedly during treatment. Particularly, the appearance of intraoral scan has made it easier to obtain digital dental models^18^.

So far, superimposition of maxillary digital models based on palatal vault region has not been compared with CBCT maxillary superimposition in adults in evaluating tooth movement. This study aims to evaluate the accuracy and reliability of 3D maxillary digital model (MDM) superimposition by comparing it with CBCT maxillary superimposition in adults. If the accuracy and validity of MDM superimposition were fully validated, we would be able to replace CBCT maxillary superimposition by radiation-free dental model superimposition to monitor the tooth movements during orthodontic treatment and to evaluate treatment effects in adults.

## Methods

### Patients

Pre- and post-treatment (T1 and T2) CBCTs and dental casts of 20 patients (5 men, 15 women) were collected among consecutive patients from the Department of Orthodontics, Peking University School and Hospital of Stomatology. Adult patients, with an average of 22.5 ± 3.1 years old, were included in this study. Exclusion criteria were patients with craniofacial abnormalities, craniofacial surgery or trauma history, CBCTs and dental casts of low quality. CBCT scans were taken by Newtom VGi (Quantitative Radiology, Verona, Italy) with the following settings: field of view, 200 × 200 mm^2^; 90 kV; 6.0 mA; scan time, 15 s; and voxel size, 0.3 mm. Dental casts were scanned by a laser scanner (R700 linear laser scanner, 3Shape Corp., Denmark) and saved as Stereo-Lithography Interface (STL) format. Informed consent forms were signed by all the patients, and the research protocol was approved by the Institutional Review Board of Peking University School and Hospital of Stomatology (IRB00001052-09010).

**Figure 1:**
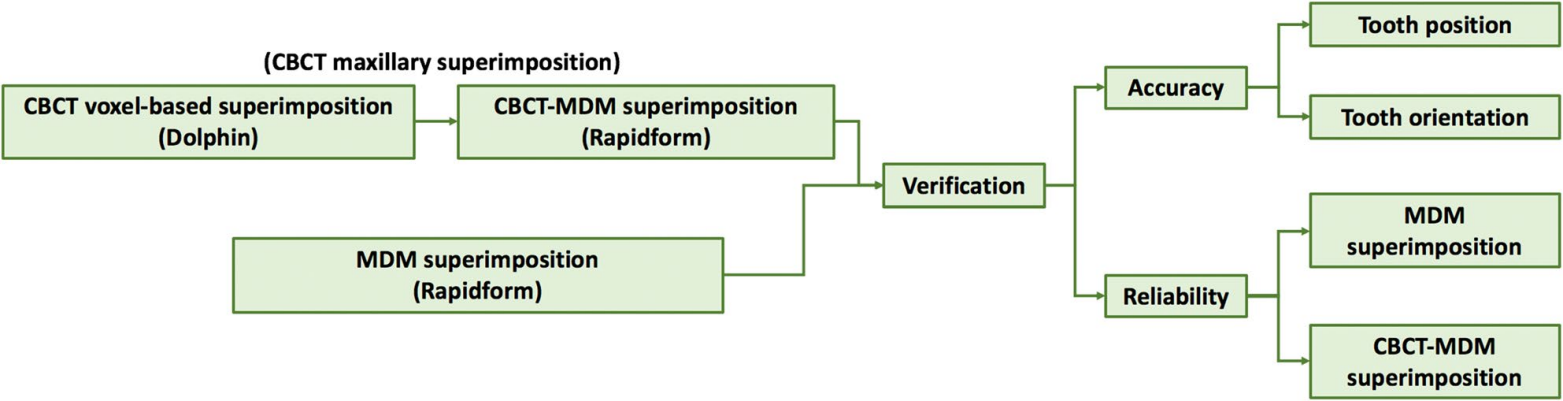
Flowchart of the study procedure. *MDM* maxillary digital model.

CBCT maxillary superimposition was composed by two procedures. Firstly, CBCT voxel-based superimposition was conducted by Dolphin software, and then pre- and posttreatment MDMs were matched with corresponding CBCT models (CBCT-MDM superimposition) using Rapidform software. MDM superimposition (palatal vault regional superimposition) were performed using Rapidform software (Fig. 1).

### CBCT voxel-based maxillary superimposition

**Figure 2:**
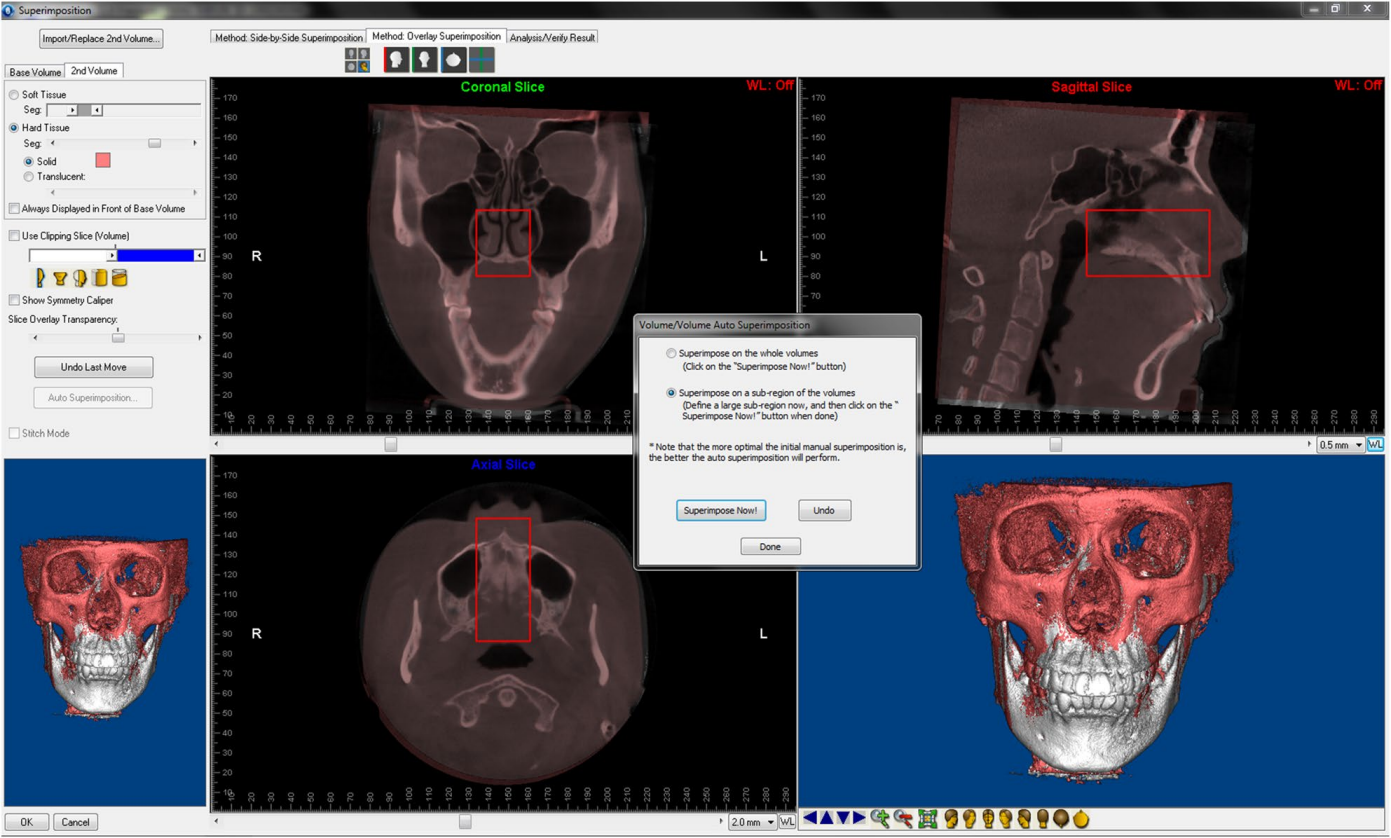
Subregion box (red) showing the maxillary area.

T1 and T2 CBCT scans were superimposed using Dolphin 11.8 Premium (Dolphin Imaging & Management Solutions, Chatsworth, CA, USA)^19^. The head position was calibrated to set the axial sections parallel to the Frankfort horizontal plane to make sure the selected superimposition region basically consistent among all the patients. Three landmarks were chosen in the Side-by-Side Superimposition tab to primarily align the two images. Voxel-based superimposition was performed on the Overlay Superimposition tab; the maxillary structures were selected by the red subregion box (Fig. 2). Oriented post-treatment CBCT was exported as Digital Imaging and Communications in Medicine (DICOM) format. The surface model mesh clearly showing the dental crown areas of the hard structures of T1 and T2 CBCT scans were exported into STL format.

### CBCT-MDM superimposition

**Figure 3:**
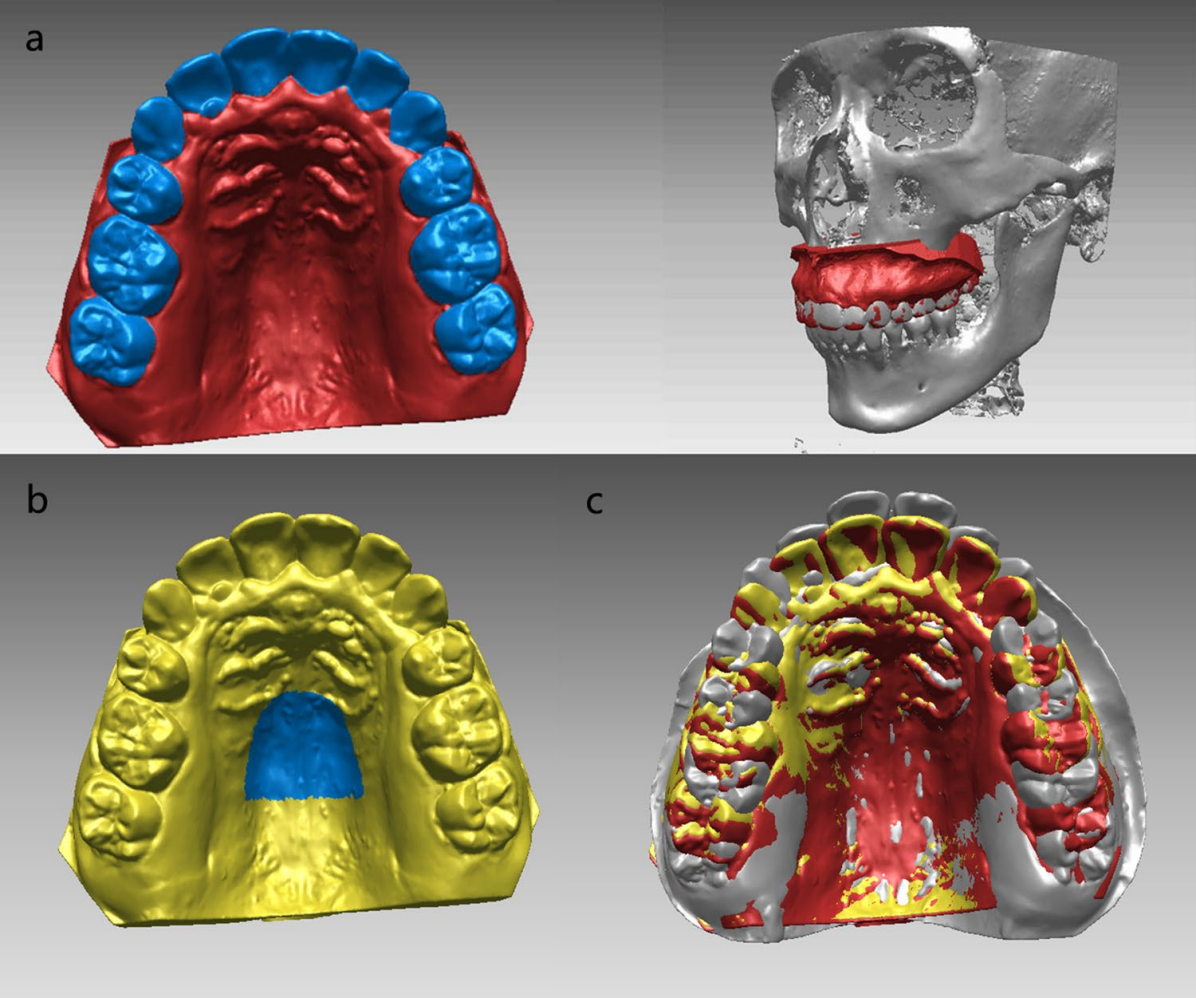
(**a**) Dental crown areas (blue area) as a reference for CBCT-MDM superimposition on T2 model (red); (**b**) palatal vault region (blue area) as a reference for superimposition of T1 (grey) and T2′ (yellow) models; (**c**) superimposed T1 (grey), T2 (red) and T2′ (yellow) models.

CBCT-MDM superimposition was conducted using Rapidform2006 (INUS Technology Inc., Seoul Korea) and dental crowns in MDMs were used for measurement instead of CBCT images. T1 and T2 maxillary digital models (grey and red) were superimposed respectively to pre- and post-treatment CBCTs by dental crown areas^20^ (Fig. 3a).

### MDM superimposition

Post-treatment digital model superimposed by the palatal vault region was recorded as T2′ model (yellow). T2′ model was initially transferred to align with the T1 model (grey). Then, T1 and T2′ digital dental models were superimposed by the palatal vault region proposed in a previous study^12^ which refers to the medial 2/3 of the third rugae and the following regional palatal vault (Fig. 3b, blue area).

### Accuracy of the MDM superimposition

Accuracy of the MDM superimposition was evaluated in two aspects using Rapidform2006, tooth position and tooth orientation.

**Figure 4:**
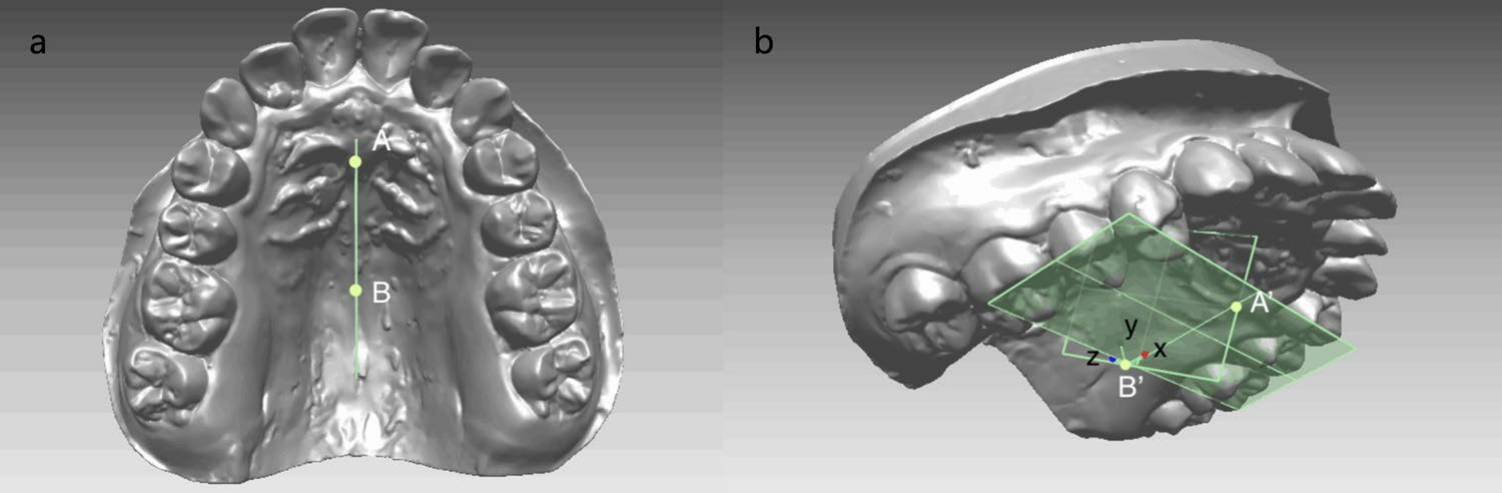
The coordinate system on T1 model.

Firstly, a coordinate system on T1 model was created (Fig. 4). Functional occlusal plane (FOP, fitted plane of the cusps of bilateral maxillary first premolars, second premolars and first molars except the distal-lingual cusp of U6s) was defined as the transverse plane. Then two points was marked on the palatal suture as point A, B (Fig. 4a), and projections of A, B on the FOP were marked as A′ and B′. B′ was defined as the origin, and B′A′ was defined as the orientation of X axis, and B′B the Y axis (Fig. 4b). The AA′BB′ composed the sagittal plane (Fig. 4b).

**Figure 5:**
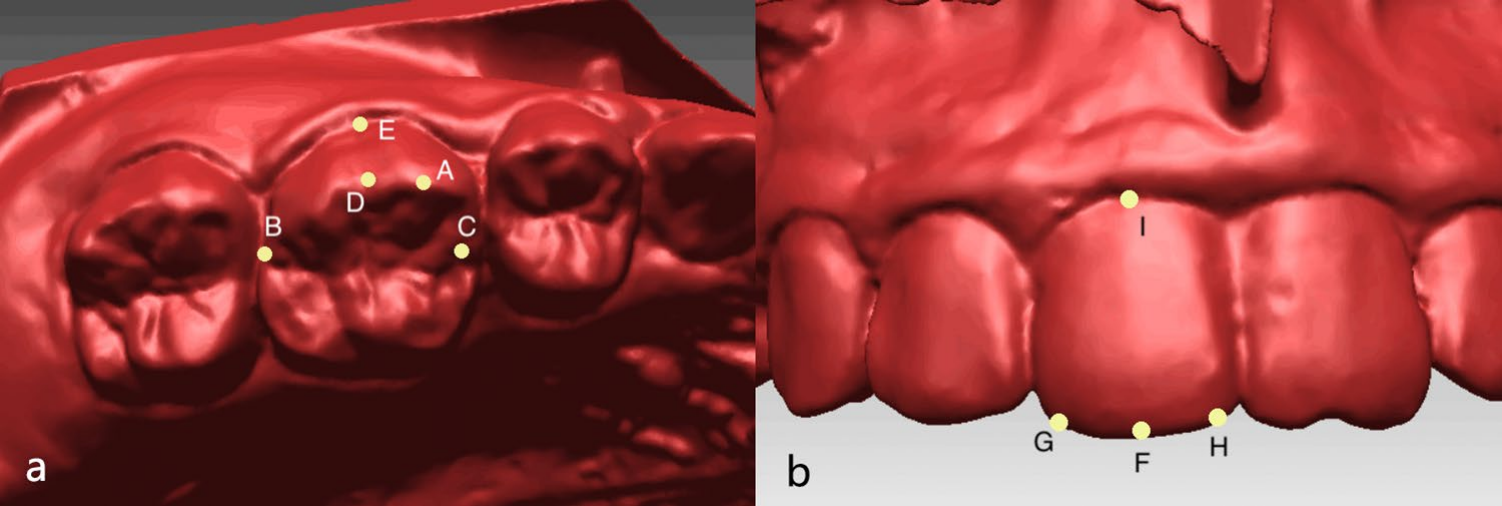
(**a**) Landmarks for U6: A. mesial-buccal cusp; B. distal point; C. mesial point; D. the most occlusal point of the buccal groove; E. the most gingival point of the buccal groove; (**b**) landmarks for U1: F. midpoint of incisal edge; G. distal point; H. mesial point; I. midpoint of gingival edge.

Tooth position of U6s and U1s respectively was represented by the three-dimensional coordinates (x, y, z) of U6’s mesial-buccal cusp (Fig. 5A) and the midpoint of U1’s incisal edge (Fig. 5F) on T2 models. Tooth orientation was represented by the angle between the reference planes and the tooth axes of U6s and U1s. The landmarks needed for definition of reference planes were shown in Fig. 5. For U6 (Fig. 5a), the mesiodistal plane of the tooth were formed by the distal and mesial points (B and C) along the occlusal central groove and their projected points on FOP. The connection of the most occlusal and gingival points (D and E) of the buccal groove was defined as the U6tooth axis. Torque of U6 was defined as the angle between the projection of U6 tooth axis onto mesiodistal plane and the normal direction of FOP^21^. Buccolingual plane of U6 was generated by the plane perpendicular to both the mesiodistal plane and FOP. Tip of U6 was defined as the angle between the projection of U6 tooth axis on buccolingual plane and the normal direction of FOP. For U1 (Fig. 5b), the mesiodistal plane were formed by distal and mesial points (G and H) along the incisal edge and their projected points on FOP. midpoint of the incisal edge and the gingival edge (F and I) was connected to represent the U1 tooth axis. Torque and Tip of U1 was generated by the same procedure as U6.

**Figure 6:**
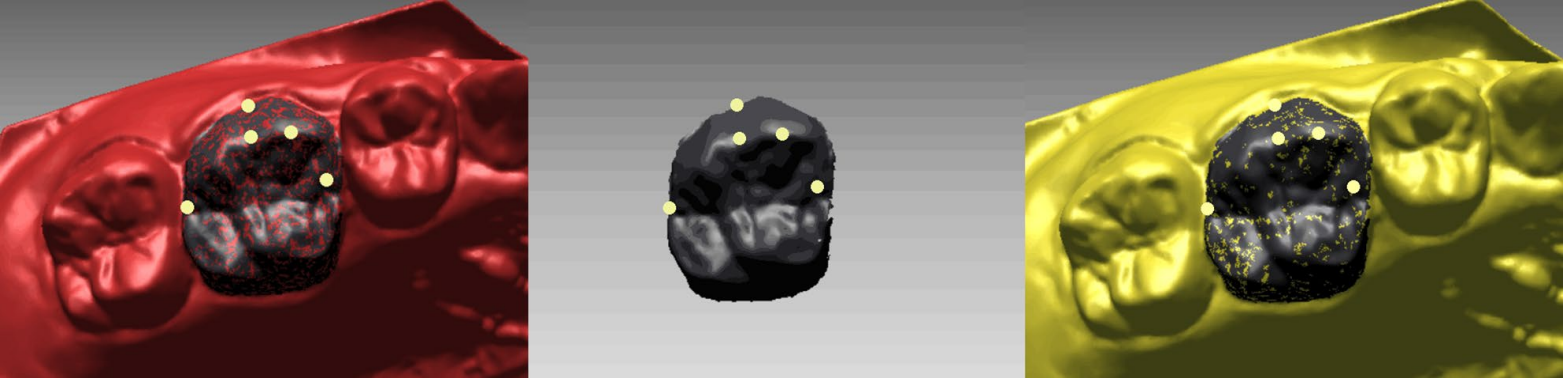
Transference of the dental crowns with landmarks from T2 (red) to T2′ (yellow) model.

We copied the crown area of U6s and U1s (Fig. 6 shows U6, the black area) on T2 model (red) and bonded the landmarks to the crown. Then the crown with landmarks was superimposed to T2′ model (yellow). This step was to exclude the errors from tooth landmarks identification. Tooth position and orientation on T2′ model was measured the same way as on T2 model.

The deviations of T2 and T2′ models were expressed by subtracting the three-dimensional tooth coordinates and the tooth angulation of T2 model from those of T2′ model.

### Reliability of MDM superimposition and CBCT-MDM superimposition

To evaluate reliability of this procedure, 10 of the 20 patients were randomly selected. MDM superimposition and CBCT-MDM superimposition were repeated by 2 trained examiners on the 10 patients, and three-dimensional coordinates (x, y, z) of the landmarks on each model were recorded. Moreover, the same measurements of the 10 patients were repeated by the first examiners 1 month later, and the coordinates on the repeated model were recorded.

### Statistical analysis

Data were analyzed by SPSS Statistics (version 23.0, IBM, IBM, Armonk, NY). To assess the accuracy of measuring tooth position and tooth orientation, one sample t-test were conducted by comparing the three-dimensional deviations of T2 and T2′ models with 0. To assess the reliability of MDM superimposition and CBCT-MDM superimposition method, intraclass correlation coefficients (ICCs) were calculated between landmark positions on T2 and T2′ models. A p-value of over 0.05 was considered statistically significant.

### Ethics approval and informed consent

The study was reviewed and approved by the Institutional Review Board of Peking University School and Hospital of Stomatology (IRB00001052-09010). All procedures performed in studies involving human participants were in accordance with the ethical standards of the institutional and/or national research committee and with the 1964 Helsinki declaration and its later amendments or comparable ethical standards. Informed consent was obtained from each patient before participation in the study.

## Results

### Accuracy of the MDM superimposition

The signed deviation in tooth position between T2 and T2′ models was shown in Table 1. There was no significant difference from zero for the three-dimensional deviation in landmark positions between CBCT maxillary superimposition and MDM superimposition except for the absolute distance between each landmark (Table 1).The average deviations in three dimensions of all U6s and U1s were all less than 0.3 mm. The absolute deviation between the landmarks were 0.62 to 0.90 mm.

**Table 1:** Signed deviation in tooth position between T2 and T2′ models.

Variable	Mean (mm)	SD (mm)	p-value
RU6-x	− 0.12	0.76	0.49
RU6-y	0.26	0.70	0.06
RU6-z	− 0.08	0.19	0.11
RU6-total	0.90	0.59	< 0.01
LU6-x	− 0.17	0.41	0.13
LU6-y	0.12	0.49	0.18
LU6-z	− 0.08	0.21	0.15
LU6-total	0.62	0.32	< 0.01
RU1-x	− 0.13	0.44	0.26
RU1-y	0.16	0.61	0.17
RU1-z	− 0.10	0.42	0.40
RU1-total	0.76	0.44	< 0.01
LU1-x	− 0.14	0.35	0.12
LU1-y	0.13	0.57	0.21
LU1-z	− 0.10	0.42	0.40
LU1-total	0.72	0.37	< 0.01

*RU6* right maxillary first molar, *LU6* left maxillary first molar, *RU1* right maxillary central incisor, *LR1* left maxillary central incisor.

-x, y, z, the signed deviation along x, y or z axis; -total, the absolute deviation between landmarks on T2 and T2′ models.

The signed deviation in tooth orientation between T2 and T2′ models was shown in Table 2. There was also no significant difference from zero for the orientations of U6s and U1s between the two superimposition methods. The average deviations were all less than 0.2°.

**Table 2:** Signed deviation in tooth orientation between T2 and T2′ models.

Variable	Mean (°)	SD (°)	p-value
RU6-tip	0.17	0.68	0.19
RU6-torque	0.11	0.81	0.41
LU6-tip	0.16	0.74	0.24
LU6-torque	− 0.16	0.74	0.46
RU1-tip	0.07	0.78	0.54
RU1-torque	− 0.07	0.73	0.81
LU1-tip	− 0.15	0.75	0.49
LU1-torque	0.03	0.69	0.70

### Reliability of the MDM superimposition and CBCT-MDM superimposition

The intra-class correlation coefficient (ICC) was calculated to evaluate the reliability (Table 3). The inter-examiner ICCs and intra-examiner ICCs were all higher than 0.99 in CBCT-MDM registration showing very high reliability of this procedure. The inter-examiner ICCs ranged from 0.85 to 0.99 and the intra-examiners ICC 0.88 to 0.99 in MDM superimposition.

**Table 3:** Intraclass correlation coefficients (ICCs) for MDM superimposition and CBCT-MDM superimposition.

	MDM superimposition		CBCT-MDM superimposition	
	Intra-examiner ICC	Inter-examiner ICC	Intra-examiner ICC	Inter-examiner ICC
RU6-x	0.99	0.99	0.99	0.99
RU6-y	0.88	0.85	0.99	0.99
RU6-z	0.96	0.98	0.99	0.99
LU6-x	0.99	0.99	0.99	0.99
LU6-y	0.91	0.93	0.99	0.99
LU6-z	0.98	0.98	0.99	0.99
RU1-x	0.99	0.99	0.99	0.99
RU1-y	0.97	0.96	0.99	0.99
RU1-z	0.93	0.87	0.99	0.99
LU1-x	0.99	0.99	0.99	0.99
LU1-y	0.97	0.97	0.99	0.99
LU1-z	0.95	0.91	0.99	0.99

## Discussion

This study validated that in adults, maxillary digital model (MDM) superimposition can replace CBCT maxillary superimposition in analyzing changes in tooth position and orientation during orthodontic treatment.

Analysis on treatment changes in tooth and bone are usually based on superimposition by stable structures. In 2-dimensional cephalometric radiograph, Björk et al.^22–24^ have established superimposition based on stable regions using metallic implants. Since its introduction in 1980s^25^, CBCT has proved to be an efficient tool in detecting problems of craniofacial structures and made it possible to superimpose on three-dimensional structures^3,4^.

It has been proved that CBCT voxel-based superimposition using Dolphin software is a precise and stable tool in application on adults^15,16,19,26,27^. However, CBCT scans are usually taken in occlusal position, in which case the occlusal surface of the teeth is invisible, and dental crown area is usually unclear in reconstructed CBCT images. On the other hand, maxillary and mandibular dental models can be separately scanned which makes it possible to accurately display the crown structures. Therefore, the MDM was superimposed onto the CBCT in order to combine CBCT’s ability to superimpose series images by bone structures and MDM’s ability to vividly display dental crowns. It showed that all the ICC values were greater than 0.99 which indicated excellent reliability in CBCT-MDM superimposition.

There are several limitations on CBCT’s use in clinical practice. Compared with MDM, CBCT expose patients on extra radiation, so it cannot be taken in a short interval or repeatedly on nonpatients. In adults, we can use superimposition on MDMs as a substitution in measurements of tooth movement. Superimposition on palatal structures were usually based on part or whole palatal vault^11,12^. A previous work by Chen et al. from our laboratory in 2011 has established palatal stable region using metallic implants, whose stability was verified in their study^12^. The study compared palatal vault regional superimposition with implant superimposition, demonstrating both good validity and reliability^12^. However, surface of the palatal vault region is soft tissue instead of rigid bone structures which makes the region more variable. In this study, we compared MDM superimposition with CBCT maxillary superimposition. The average signed deviations in three dimensions between the two post-treatment models (T2 and T2′ models) by MDM superimposition and CBCT maxillary superimposition respectively were all less than ± 0.3 mm and ± 0.2°, which is statistically insignificant from zero and could be recognized as clinically acceptable. The absolute deviation of the landmarks between the two superimposition methods was less than 1 mm. As the ICCs were remarkably high for CBCT maxillary superimposition, the main error must have come from MDM superimposition. In this study, we also repeat the MDM superimposition procedure and as a result, all the ICC values were larger than 0.85, which was not as good as CBCT maxillary superimposition. The main reason is that MDM superimposition is a soft tissue-based method and only a specific region can be selected as the stable region. However, the boundary of the stable region is not distinct, which may diminish the reliability.

Compared with CBCT maxillary superimposition, the MDM superimposition, to a large extent, depends on the quality of dental casts. Bubbles and defects in dental casts, especially in palatal vault region, may greatly influence the result of palatal region superimposition^8^. In recent years, the introduction of intraoral scan has simplified the obtaining of digital dental models. With a trueness of over 200 μm^28^ and a precision of over 1000 μm^29^, intraoral scan can accurately and reliably reproduce the maxillary and mandibular teeth and occlusion relationship in real time without bubbles or defects coming from operation. Furthermore, more comfortable and convenient oral scanning makes it possible to record the changes in tooth and occlusion relationship at any frequency the orthodontists want. If combined with MDM superimposition, we may realize chair-side superimposition which may show the patients the changes of their teeth at every visit.

On the whole, the MDM superimposition method was accurate and reliable for maxillary superimposition in adults to serve as an efficient method to evaluate teeth movement. It provides a basis for a radiation-free approach to monitor and assess the tooth movements during orthodontic treatment in adults. With MDM superimposition, we will be able to follow treatment changes step by step and adjust treatment plans when necessary which conforms the principle of the upcoming era of ‘monitored orthodontic treatment’. With the development of intra-oral scan, digital dental models can be acquired easily and superimposed accurately, which makes remote monitoring possible. Remote monitoring is important in modern society where people flow frequently wearing appliance. Furthermore, this superimposition method makes it possible for the dentists to take full advantage of the precious clinical materials of the patients whose treatment was completed before the appearance of CBCT.

## Conclusion

Compared with CBCT maxillary superimposition, maxillary digital model (MDM) superimposition proved to be an efficient, accurate and reliable method to evaluate tooth movements in adults, with a similar accuracy but a slightly lower reliability than CBCT maxillary superimposition. We believed that 3D MDM superimposition was able to replace CBCT maxillary superimposition in evaluating maxillary tooth movement.

## Data Availability

All data generated or analysed during this study are included in this published article.
